# Effects of colchicine on major adverse cardiac events in next 6-month period after acute coronary syndrome occurrence; a randomized placebo-control trial

**DOI:** 10.1186/s12872-021-02393-9

**Published:** 2021-12-07

**Authors:** Mehdi Akrami, Peyman Izadpanah, Mehdi Bazrafshan, Unes Hatamipour, Navid Nouraein, Hamed Bazrafshan Drissi, Alireza Manafi

**Affiliations:** 1 Cardiovascular Department, Shiraz University of Medical Sciences, Shiraz, Iran; 2 Cardiovascular Research Center, Shiraz University of Medical Sciences, Shiraz, Iran; 3 Student Research Committee, Shiraz University of Medical Sciences, Shiraz, Iran

**Keywords:** Coronary artery disease, Colchicine, Acute coronary syndrome, Inflammation

## Abstract

**Background:**

Cardiovascular disease in particular acute coronary syndrome (ACS) is remained one of the most cause of morbidity and mortality, annually. Considering inflammatory pathway of atherosclerosis, colchicine as an anti-inflammatory drug is introduced to be effective in pathogenesis, prognosis and mortality rate of these patients. So in order to find out the effects of this drug we conducted this trial to know whether it reduces major adverse cardiac events (MACE) in ACS patients or not.

**Methods:**

In a prospective randomized double-blinded placebo-controlled trial, we enrolled ACS patients (40–70 years) with recent ST-segment elevation myocardial infarction (STEMI) or NSTE-ACS diagnosed by coronary angiography and managed with either medical therapy or percutaneous coronary intervention. Patients were assigned to two groups either receiving colchicine 0.5 mg daily or placebo for 6 months. Both groups simultaneously received standard medical therapy as accessible guidelines. MACE occurrence consists of decompensated heart failure, ACS, stroke and survival rate compared between two groups.

**Results:**

A total of 249 patients were recruited between October 2019-March 2020 with mean age of 56.89 ± 7.54, 69.5% males; 120 assigned to the colchicine group and 129 assigned to the placebo group. Over the 6 months’ period, 36 MACE occurred that were 8 events in the colchicine group compared with 28 events in the placebo group experiencing the event (*P* = 0.001). All of four deaths in the colchicine group and two in the placebo group were due to cardiovascular events. Evaluating adverse effects, gastrointestinal symptom was the most with the rate of 15 (12.5%) in the colchicine group and 3 (2.5%) in the controls. (*P* = 0.002).

**Conclusion:**

The addition of colchicine to standard medical therapy in ACS patients significantly reduces MACE occurrence and improves survival rate over the time.

## Introduction

In spite of multilateral attempts managing primary and secondary preventive strategies, cardiovascular diseases have been the main cause of mortality in recent decades [1, 2]. Accordingly, paying attention to new and effective preventive and curative plans has been turned into an emerging issue. Inflammation, as a multidirectional stream in the human body with chronic effects, plays a central and pivotal role in all stages of atherosclerosis pathogenesis [3–5]. Moreover, cardiovascular risk factors such as dyslipidemia, hypertension, dysglycemia, opium use and smoking impair endothelial vasculature. As a result, an inflammatory response is triggered, thereby contributing to the release of cytokines and other inflammatory mediators which trigger further destruction [6, 7]. Likewise, it was Canakinumab Anti-inflammatory Thrombosis Outcome Study (CANTOS) trial that revealed the inhibition of interleukin (IL)-1β, as an important inflammatory cytokine, by Canakinumab and its association with reduced cardiovascular events [8].

An old drug with newly presented anti-inflammatory potentials, colchicine has transformed the treatment of inflammatory-based cardiovascular events [9]. It was first extracted from autumn crocus and was introduced for many centuries ago. The main pathways, which are ascribed to colchicine, are tubulin polymerization and microtubule generation inhibition which affects inflammatory chemokine and inflammasome [10, 11]. These molecular pathways ultimately bring about the downstream of inflammatory cascades and innate immunity which are the main pathogenesis of thrombotic events in patients with acute coronary syndrome (ACS) [5].

Although some reviews such as Malik et al. [12] have questioned the positive role of anti-inflammatory therapies, specially colchicine, in treatment of acute coronary syndrome, some others have strongly proven the significantly reduced cardiovascular events in ACS patients using 0.5 mg/day in addition to standard preventive therapies. In Low Dose Colchicine for Secondary Prevention of Cardiovascular Disease (LoDoCo) and Colchicine Cardiovascular Outcomes Trial (COLCOT) trials, low dose colchicine plus standard secondary prevention therapies compared with standard medical therapies alone and concluded that this regimen has more reduction rates in cardiovascular complications in ACS patients [13, 14]. These findings are in line with those of other previous studies which report the efficiency of low-dose colchicine therapy. It modifies coronary plaques independent of substantial low-density lipoprotein reduction and high-dose statin intensification therapy [15]. Also, another survey demonstrated that colchicine therapy is associated with reduced inflammatory cytokines and limited infarct size in patients who underwent percutaneous coronary intervention (PCI) after an episode of ST-segment elevation myocardial infarction (STEMI) [16, 17].

This study was conducted to evaluate the effect of short-term, low-dose colchicine therapy, along with the standard medical therapy in approved ACS patients within a period of six months after a cardiac event.

## Methods

### Trial design and population

The present study was a prospective double-blinded placebo-controlled randomized trial which included a total of 361 ACS patients (40–70-year-old adults) who visited a tertiary healthcare heart hospital affiliated to Shiraz University of Medical Sciences from October 2019 till March 2020. All the patients underwent coronary angiography (explained as ≥ 50% luminal stenosis in any epicardial vessel of ≥ 2.5 mm luminal diameter) and were managed with either PCI or medical therapy. ACS patients were included as three groups with either elevated troponin or ECG changes: unstable angina (UA), non-ST segment elevation myocardial infarction (NSTEMI) and STEMI. Patients with unstable angina had typical chest pain and ECG alteration without elevated troponin and elevated ST segment. NSTEMI patients were those who had elevated troponin without ST-segment elevation, and STEMI patients exhibited ST elevation in ECG and had a positive troponin test. The exclusion criteria were any history of long-term colchicine use or hypersensitivity to it, moderate renal dysfunction (glomerular filtration rate ˂50), hepatic dysfunction, thrombocytopenia, leukemia, left ventricular ejection fraction ˂30%, surgical revascularization and lactation or the risk of pregnancy.

We randomly assigned patients with a 1:1 aspect ratio to receive either colchicine (at a single dose of 0.5 mg once daily), plus standard medical therapy and standard medical therapy plus placebo. We used a computer-based program with block randomization protocol as well as the method of block size factor obtained from the study investigators. Preparing packages of study medications was performed by an independent packaging team that was not involved in the rest of the study. All the investigators, patients and follow-up team members were unaware so as to devise a non-biased controlled study, whereas unbinding was performed in exceptional cases of unwell patients wherein the knowledge of treatment allocation was necessary The present study was also registered at (irct.ir) with a registration number of IRCT20201117049420N1.

### Trial procedures

All the patients obtained standard medical treatments in accordance with the local ACS management guidelines. Patients who were placed in the intervention group received 0.5 mg/daily colchicine, whilst the control group members were given placebo tablets. Screening, randomization, and administration of colchicine were performed on the first day of ACS occurrence. Considering shape, size, color and packaging, placebo tablets were the same as colchicine. The follow-up was carried out seven days after the patients were discharged from hospital by means of structured telephone interviews. Moreover, the follow-up process continued monthly to control patients` tolerability, adherence to study and major adverse cardiac events (MACE). For further confidence, all the patients were checked every two months by investigators. This follow-up was performed by investigators who were unaware of the study groups' allocation. We kept the patients under scrutiny for a period of six months after ingestion of first colchicine or placebo tablets. During these six months, the blinding of the research team to outcome of the study was maintained.

### Trial outcomes

The primary outcomes were death from any cause, non-cardio-embolic ischemic stroke, hospital admission due to typical chest pain (UA, STEMI/NSTEMI), urgent need for revascularization and decompensated heart failure. The secondary end points consisted of the components of the primary efficacy end point; a combination of hospitalization for chest pain, death from cardiovascular causes, resuscitated cardiac arrest, myocardial infarction, or stroke; and total mortality in time-to event analyses.

### Statistical analysis

The collected data was placed into the statistical package for social sciences version 18 (SPSS Inc., Chicago, IL, USA). Descriptive statistics including mean (standard deviation) for quantitative variables and number (%) for qualitative variables were used to describe the data. Data analysis was conducted using independent sample T-test and Chi-square test. The use of independent T makes it possible to measure the average of the parameters before and after, as well as the difference between the changes. Furthermore, *t*-test was used to compare the mean of parameters in each of the two groups. The Chi-square test was also employed to make a comparison between the qualitative variables and qualities for the two groups. Normality of distributions was evaluated using the frequency histograms and the Shapiro-Wilkes test. The primary outcome was a time to event analysis via the logrank test. A sensitivity analysis accounted for multiple correlated events within in an individual by using a Cox regression with group assignment as the independent variable, clustering over individual and reporting robust standard errors. Results of this study are reported as hazard ratios (HR) with 95% confidence intervals (95%CI). A *P*-value less than 0.05 (two-tailed) was regarded as statistically significant.

### Sample size justification

Given the goals and the type of this study, the citation was based on the previous studies [9] in this field while taking into account the assumptions: the error of 5%, the power of 80%, the effect size of about 40% or the risk ratio of about three percent in the two groups, and the ratio of one to one in two groups were all measured by means of the following formula:

$$n = \frac{{2\overline{p}(1 - \overline{p})(z_{{1 - {\raise0.7ex\hbox{$\alpha $} \!\mathord{\left/ {\vphantom {\alpha 2}}\right.\kern-\nulldelimiterspace} \!\lower0.7ex\hbox{$2$}}}} + z_{1 - \beta } )^{2} }}{{(\partial )^{2} }}$$

Considering a five percent drop in total amount obtained from the computations, a total of at least 120 patients in each group and a total of 240 patients are needed. It should be noted that the sampling method is purposeful sampling method which is easily based on the purpose of the study. Accordingly, the investigators were present at the time of the study and started sampling from accessible referral patients to obtain the total sample size.

### Trial oversight

This study was approved by the institutional review board of Shiraz University of Medical Sciences and won the approval of the Ethical Committee (IR.SUMS.MED.REC.1398.409). Likewise, the present study was also registered at (irct.ir) with a registration number of IRCT20201117049420N1. It should be noted that written informed consents were obtained from all the participants in the study. The patients’ MACE was evaluated by two independent cardiologists who were blinded to (unaware of) the treatment allocation.

## Results

### Patients

**Fig. 1:**
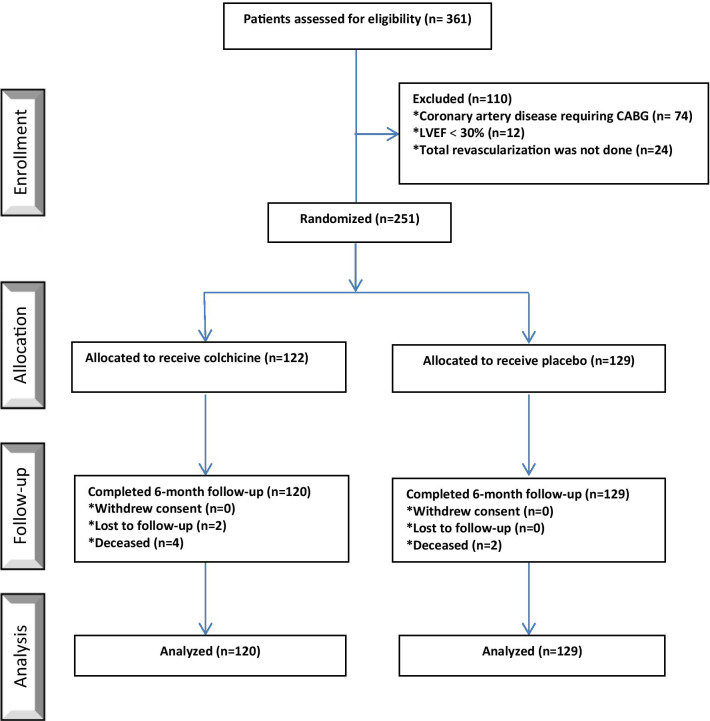
Consort flow diagram of the trial. CABG, coronary artery bypass graft LVEF, left ventricular ejection fraction

Subjects’ enrollment in the trial started in October 2019 and ended in March 2020. After exclusion, a total of 249 patients were selected through randomization (122 and 129 subjects were assigned to colchicine and the placebo groups, respectively) and were then kept under study for a 6-month period. The participants’ mean age was 56.89 ± 7.54 with males comprising 69.5% of participants. In colchicine group, it was found that at the end of the enrollment, 15 (12.5%) patients had a history of gastrointestinal adverse effects caused by colchicine use in compare with 3 (2.5%) in placebo group (diarrhea, *P* = 0.002). It should, however, be noted that only two of the subjects in study group did not tolerate the effects, thereby were excluded from the study. It is noteworthy that four and two patients in colchicine and placebo group, respectively were deceased during this follow-up period (*P* value = 0.35); yet considering death as a factor of MACE, their information was incorporated into our database. Details regarding the disposition of the patients are provided in Fig. 1 as CONSORT flowchart. All the participants in both groups received antiplatelet drug (also known as a platelet agglutination inhibitor or platelet aggregation inhibitor), statin, beta blocker and angiotensin-converting-enzyme inhibitor as a standard ACS medical treatment. Patients’ data regarding their past medical history in two groups is recorded and available in Table 1.

**Table 1:** Baseline characteristics of the patients

Characteristics	Colchicine (n = 120)	Placebo (n = 129)	*P* value
Age, year (mean ± SD)	56.9 ± 7.56	56.89 ± 7.45	0.993
Male sex, no. (%)	86 (71.7)	87 (67.4)	0.939
Hypertension, no. (%)	52 (43.3)	59 (45.7)	0.703
Diabetes, no. (%)	27 (22.5)	32 (24.8)	0.669
Current smoking or opium use, no. (%)	52 (43.3)	49 (38.0)	0.39
Hyperlipidemia, no. (%)	37 (30.8)	36 (27.9)	0.78
History of myocardial infarction, no (%)	10 (8.3)	12 (9.3)	0.788
History of PCI, no. (%)	16 (13.3)	20 (15.5)	0.627
History of CABG, no. (%)	4 (3.3)	3 (2.3)	0.714
History of heart failure, no. (%)	4 (3.3)	5 (3.9)	0.819
History of TIA or CVA, no. (%)	5 (4.2)	4 (3.1)	0.653
Admission diagnosis			
STEMI	64 (53.3)	64 (50.4)	0.557
NSTEMI + UA	56 (46.7)	65 (49.6)	0.642
UA	41 (34.2)	45 (34.8)	0.905
NSTEMI	15 (12.5)	20 (14.8)	0.496
Number of patients underwent PCI	104	111	0.887
PCI to culprit vessel			
LAD	54	62	0.628
LCX (OM)	20	23	0.808
RCA	23	21	0.551
Ramus	2	2	0.942
PDA	5	3	0.41

PCI, percutaneous coronary intervention; CABG, coronary artery bypass graft; TIA, transients ischemic attack; CVA, cerebrovascular accident; STEMI, ST segment elevation myocardial infarction; NSTEMI, non-ST segment elevation acute coronary syndrome; UA, unstable angina; LAD, left anterior descending; LCx, left circumflex; OM, obtuse marginal; RCA, right coronary artery; PDA, posterior descending artery

### Clinical efficacy end points

**Fig. 2:**
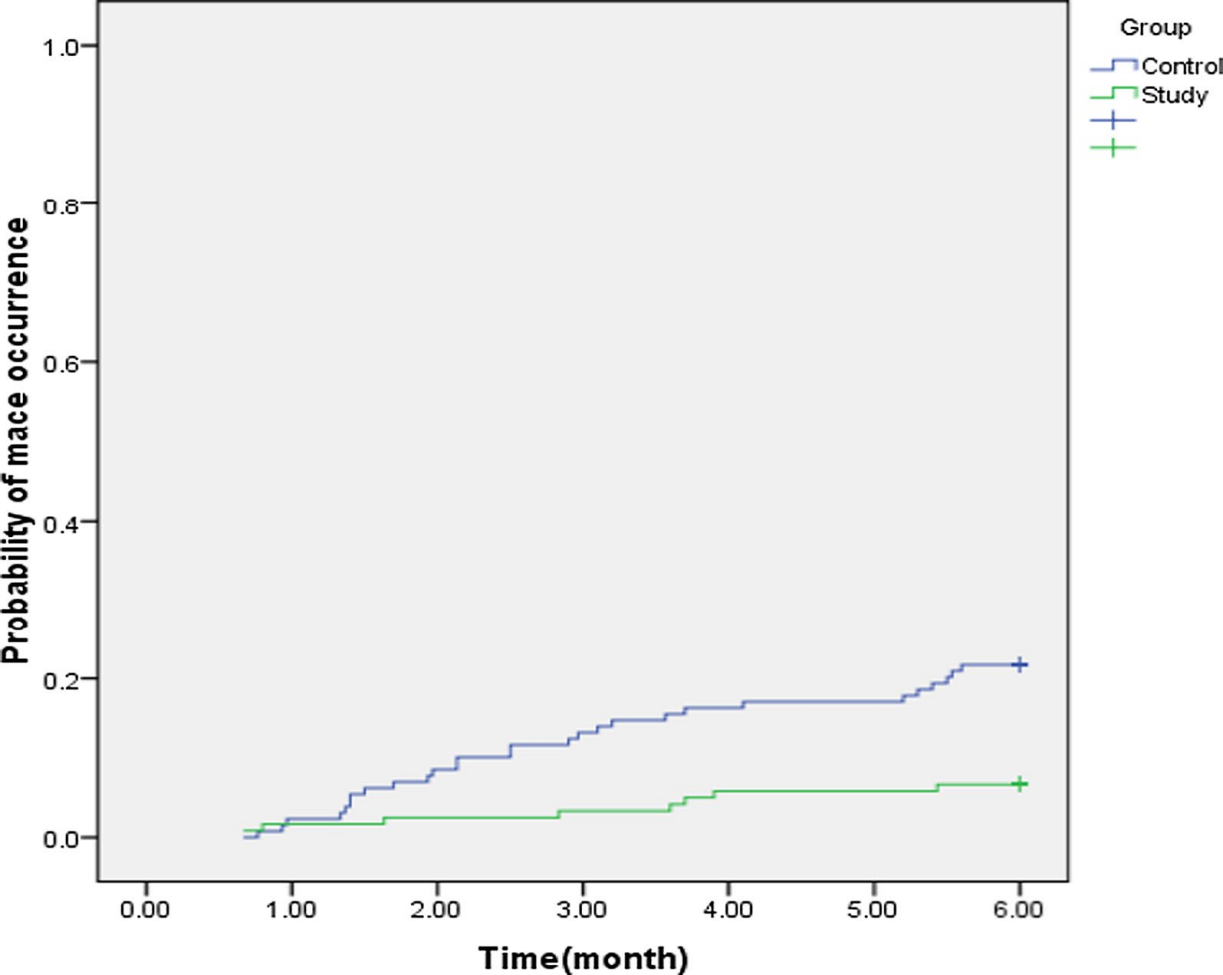
Probability of MACE occurrence in study and control group over time

At the end of the study, and after a 6-month period we found significantly lower rates of MACE in colchicine group than in placebo (8 [6.7%] *vs.* 28 [21.7%]). In a post hoc analysis (Fig. 2) of the primary outcomes (MACE), there was found significantly lower rates of events in favor of colchicine over the 6-month follow-up period (6.7% vs. 21.7%, Hazard Ratio [HR] 1.64, 95%CI 1.31–2.05, *P* = 0.001).

Based on Table 2, the most common event seen in two groups were unstable angina and NSTEMI, respectively. There was not any occurrence of stroke or TIA in all the patients in both groups, and all the causes of death were cardiovascular.

**Table 2:** Major clinical end points (intention-to-treat population)

Endpoint	Colchicine	Placebo	Hazard ratio (95%CI)	*P* value
Total MACE	8 (6.7)	28 (21.7)	3.52 (1.60–7.74)	0.001
ACS	4 (3.3)	25 (19.4)	1.82 (1.49 – 2.23)	< 0.001
STEMI	0	3 (2.3)	1.95 (1.72 – 2.20)	0.093
NSTEMI	2 (1.7)	8 (6.2)	1.58 (1.13 – 2.20)	0.069
UA	2 (1.7)	14 (10.9)	1.77 (1.41 – 2.22)	0.003
DHF	0	1 (0.8)	1.93 (1.71 – 2.18)	0.334
Death				
any cause	4 (3.3)	2 (1.6)	0.63 (0.20 – 1.99)	0.359
cardiovascular	4 (3.3)	2 (1.6)	0.63 (0.20 – 1.99)	0.359

Cox regression model clustered over multiple events with an individual and adjusted for group

assignment.ACS, acute coronary syndrome; NSTEMI, non-ST segment elevation acute coronary syndrome; UA, unstable angina; DHF, decompensated heart failure

Also we separately compared STEMI and NSTE-ACS patients between two groups regarding MACE occurrence and found them significantly in lower rates in favor of colchicine group. (*P* = 0.009 and *P* = 0.029, respectively). Moreover, the relationship between the first diagnosis and the occurrence of MACE in the two groups was investigated, and the data are shown in Table 3.

**Table 3:** The relationship between first diagnosis and endpoints in two groups

First diagnosis	MACE occurrence			
	Colchicine	Placebo	Hazard ratio (95%CI)	*P* value
ACS	8 (6.7)	28 (21.7)	3.750 (0.810–17.370)	0.091
STEMI	5 (4.1)	15 (11.6)	4.667 (0.457 – 47.629)	0.194
NSTEMI	2 (1.6)	8 (6.2)	1.688 (0.201 – 14.194)	0.63
UA	1 (1)	5 (3.9)	–	–

In the main sensitivity analysis, HR was 3.52 (95%CI 1.60–7.74, *P* = 0.002), in a time to endpoint event Cox regression with group as the independent variable. This result remained stable in another sensitivity analysis (HR 3.51, 95%CI 1.60–7.72, *P* = 0.002) when it was adjusted for age, sex, diabetes status, hypertension, hypercholesterolemia, previous MI, and smoking status, and clustered over individual. Both models satisfied the proportional hazards assumption. A comprehensive summary of the causes of death is listed in Table 4. All of deaths in colchicine group occurred when patients were receiving colchicine during their treatment course. We also compared the results between PCI and non-PCI patients in each group in terms of the correlation with MACE occurrence, but there was no significant difference in either group (*P* = 0.35 in the colchicine group and *P* = 0.54 in controls). The data were similar comparing culprit-only PCI and PCI on all stenosis vessels in each group (*P* = 0.16 in the colchicine group and *P* = 0.70 in controls). Indeed, biomarkers such as white blood cells, troponin, liver function test, blood urea nitrogen, etc. were compared between the two groups, and the data are shown in Table 5.

**Table 4:** Summary of causes of death

Patient number	Treatment group	CV or Non CV Death	Early discontinuation (within first 30 days)	Clinical information
1	Colchicine	CV	NO	Unconscious collapse with cardiac arrest and impression of anterior wall STEMI. CPR performed but unable to be
				Resuscitated. Prior angiogram demonstrated SVD that PCI was done on LAD
2	Colchicine	CV	NO	Found dead at home following rapidly transfer to hospital. Prior angiogram demonstrated 2VDthat PCI was done on ramus; and40% mild RCA lesion
				Medically managed
3	Colchicine	CV	NO	Found dead at home by family at morning
4	Colchicine	CV	NO	Admitted in hospital with impression of anterior wall STEMI and cardiogenic shock but developed with cardiopulmonary arrest before coronary angiography. Prior angiogram(after inferior STEMI)demonstratedpatent LAD stent that PCI was done on occluded RCA
5	Placebo	CV	NO	Developed with severe CP at a party following cardiopulmonary arrest.CPR performed but unable to be Resuscitated.In prior angiogram PCI on LAD was done and he had also a non-significant RCA lesion
6	Placebo	CV	NO	Developed with cardiopulmonary arrest at home.CPR performed by emergency team but unable to be Resuscitated. In prior admissionprimary PCI on LAD was done

STEMI, ST segment elevation myocardial infarction; CPR, cardiopulmonary resuscitation; SVD, single vessel disease; LAD,left anterior descending; PCI,percutaneous coronary intervention;RCA, right coronary artery; CP, chest pain

**Table 5:** Biomarkers in two study groups

Biomarkers	Colchicine			Placebo		
	(n = 120)			(n = 129)		
	Mean ± SD, Number (%)	Correlations with MACE	*P* value	Mean ± SD, Number (%)	Correlations with MACE	*P* value
WBC	6210 ± 1357.60	0.064	0.49	6156.59 ± 1338.425	0.054	0.541
Lymphocyte	2206.67 ± 307.26	0.073	0.427	2189.92 ± 299.96	0.035	0.698
Neutrophil	3625.83 ± 857.83	0.028	0.762	3596.90 ± 834.41	0.023	0.793
Troponin	79 (65.8)	0.122	0.184	84 (65.1)	0.149	0.093
TG (mg/dl)	188.91 ± 46.46	0.106	0.249	198.34 ± 58.20	0.145	0.101
Cholesterol (mg/dl)	197.93 ± 58.73	0.063	0.496	188.76 ± 53.368	0.113	0.204
LDL-c (mg/dl)	146.43 ± 28.92	0.081	0.379	150.44 ± 27.98	0.132	0.137
HDL-c (mg/dl)	40.42 ± 5.58	0.074	0.42	39.09 ± 5.35	0.169	0.056
BUN (mg/dl)	20.23 ± 6.02	0.168	0.067	20.59 ± 4.97	0.153	0.084
Cr (mg/dl)	1.16 ± 0.21	0.166	0.07	1.13 ± 0.21	0.111	0.21
SGOT (mg/dl)	29.45 ± 6.05	0.131	0.153	30.31 ± 5.94	0.025	0.781
SGPT (mg/dl)	29.04 ± 4.85	0.145	0.114	28.45 ± 4.94	0.115	0.196
ALP (mg/dl)	174.55 ± 41.53	0.121	0.19	180.29 ± 44.26	0.048	0.589
EF (%)	49.79 ± 2.78	0.019	0.834	50.30 ± 2.68	0.146	0.099

WBC, white blood cell; TG, triglycerides; LDL-c, low density lipoprotein cholesterol; HDL-c, high density lipoprotein cholesterol; Cr, creatinine; SGOT, serum glutamic oxaloacetic transaminase; SGPT, serum glutamic pyruvate transaminase; ALP, alkaline phosphatase; EF, ejection fraction

**Fig. 3:**
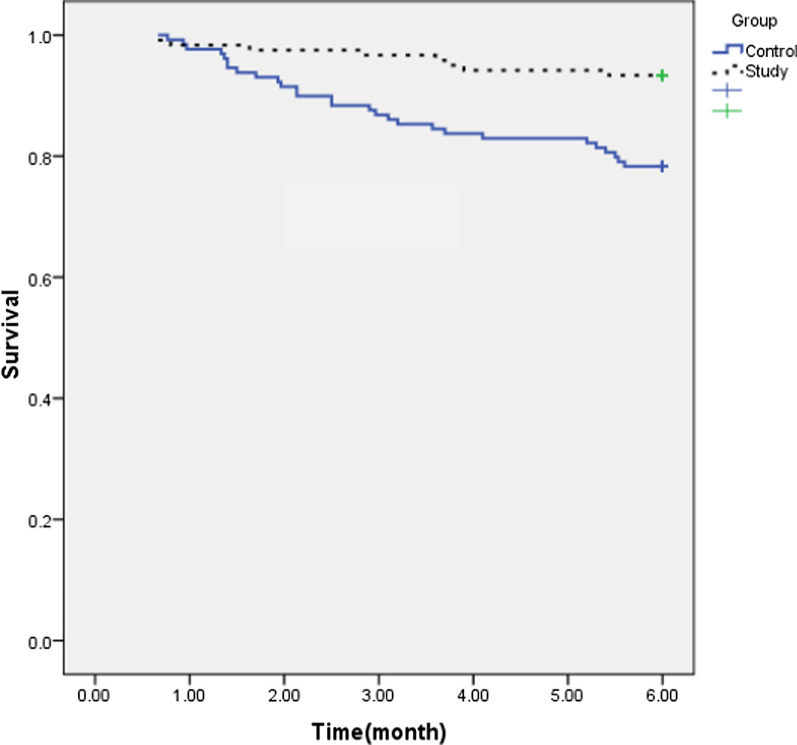
Kaplan –Meier survival curve for study and control group

In a post hoc analysis of the composite endpoint using all the occurred deaths, over the 6- month follow-up, there was a significant reduction in events in favor of colchicine (*P* = 0.001). Kaplan –Meier survival curve is presented in Fig. 3.

## Discussion

This trial has showed that the addition of low-dose oral colchicine to standard medical regimen in ACS patients could be effective such that it can significantly reduce major adverse cardiac events such as decompensated heart failure, ACS and all cardiovascular causes of death which are considered as primary composite outcomes in a 6-month period after a cardiac event. Also, MACE occurrence in STEMI and NSTEMI participants who received colchicine were at lower rates than those of the placebo group. Survival rate also were higher in colchicine group.

Besides standard antiplatelet and statin therapy in ACS patients, patients commonly are at a high risk of secondary cardiovascular events due to disregarding inflammatory pathways. Diseased vessels with atherosclerotic walls are strongly prone to injury and plaque instability [18, 19]. Basically, a recently published study as CANTOS trial has demonstrated that Canakinuma-targeting inflammatory pathway in ACS patients presented with elevated high sensitivity C-reactive protein (hs-CRP) is effective when acting as IL-1 β antagonist [8]. Although this drug showed beneficial anti-inflammatory actions to reduce recurrent cardiovascular events, it could not achieve global popularity for use due to its cost, lack of favorable effect and increasingly high rate of fatal infections seen in this study. On the other hand, colchicine with well-established accessibility and lower costs could find its place in cases of inflammatory-base disease.

Additionally, given its with broad anti-inflammatory action, colchicine genetically acts as a cytoskeletal microtubules-disassembling agent and consequently blocks co-localization with NLRP3, thereby leading to prevention of inflammasome complex assembly and activation [16, 20, 21]. Considering these cellular mechanisms of action, colchicine disrupts microtubules with mitotic effect and suppresses upregulation of IL-1β and IL-6 to play its anti-inflammatory role as well [22, 23]. Although prescription of 1/mg daily colchicine for 30 days would not decrease inflammation and hs-CRP in patients with acute coronary syndrome or acute ischemic stroke in Raju et al. [24] study, in yet another survey, Martinez et al. demonstrated a significant reduction of level of inflammatory cytokines such as IL-1β, IL-18 and IL-6 after a short-term administration of colchicine in ACS patients [16].

The Australian COPS Randomized Clinical Trial coordinators recruited 795 ACS patients in a multicenter survey to evaluate cardiovascular outcomes followed by receiving colchicine for a 12-month period [25]. After prescription of 0.5 mg colchicine twice daily for a month followed by 0.5 mg daily for 11 months, they did not find any significant reduction of cardiovascular events in colchicine group than in the placebo group. In contrary, although we followed the patients for a shorter period in the present study than that study with continuously lower doses of colchicine prescription, a receivable reduction of MACE was observed in ACS patients who received colchicine compared with placebo (8 vs. 28, *P* = 0.001, HR: 3.52, *P* = 0.002). In our study, of six cardiovascular overall deaths, four belonged to colchicine group, and two death cases occurred in placebo. However, Ton et al. reported significantly higher rate of all-cause deaths than controls. Their cause of death was sepsis in 4 of 5 patients that could be important in colchicine receiving group (all deaths were in the first 30 days of study). Our data about death rate is similar to their study. However, there was a great difference; that is, it was revealed that all deaths were caused by cardiovascular factors without any sepsis.

Most of our primary endpoints as MACE were associated with ACS occurrence with a great proportion of UA significantly observed in placebo group. STEMI, NSTEMI and DHF were also seen in controls more frequently than in study group. Vaidya et al. [26] worked on publications reviewing the anti-inflammatory role of colchicine and its role on ACS complications. Although, they explained its role of inhibiting NOD-like receptor protein 3 inflammasome complex, its prescription generally in all ACS patients has been depended on further investigation. With regard to The Low Dose Colchicine after Myocardial Infarction (LoDoCo-MI) study [27], it was found that the administration of low dose colchicine did not contribute to the reduction of hs-CRP which is independent of aspirin and statin in patients with coronary artery disease. Yet, it was shown that the incidence of non-cardio embolic ischemic stroke, ACS and out-of-hospital cardiac arrest in patients with stable coronary disease who received colchicine 0.5 mg daily was significantly lower than controls. It seems that a decreased level of hs-CRP in ACS patients who received colchicine even in lower doses, is independent of the improvement in cardiovascular event occurrence.

Considering side effects, gastrointestinal involvement is the most common complications of colchicine prescription in any dose or any diseased participant. Tong et al. [25] and Tardif et al. [13] reported higher frequency of diarrhea as the commonest gastrointestinal symptom observed in patients who received colchicine than controls without any significant correlation in separate studies. In our study, out of 15 patients that developed diarrhea, two patients left the study due to drug intolerance. In placebo group three patients had diarrhea that was significantly lower than study group.

Similar to Australian COPS RCT study and Vida et al. study [15], we conducted our study not only on STEMI patients but also on NSTEMI participants to show that beneficial effects of colchicine in all ACS patients are accessible. Further studies in the future are to be carried out to evaluate the safety of colchicine in ACS population, in particular concerning the improvement in survival rate of patients.

## Limitations

Through enrollment in patients’ laboratory data such as hs-CRP to the study, we might evaluate colchicine effects on hs-CRP levels and its correlation with reduction in MACE. On the other hand, in addition to the anti-inflammatory role of colchicine, it is presumed that it can have an increasing risk of infection in some studies. The cohort study by Tsai et al. [28] on a population discovered higher rates of pneumonia in gout patients who received colchicine compared to those who did not receive (HR = 1.42). Yet, a recent study with a review of 35 RCTs, along with a total of 8659 pooled participants demonstrated no significant difference in infectious events in the colchicine group compared to non-colchicine group (0.4% vs. 2.1%) [29]. Therefore, according to these equivocal data, it might be beneficial to evaluate infectious events and compare those between the two groups. Indeed, due to limitations in our catheterization laboratory, PCI was only performed on culprit vessels as the primary PCI and we did not perform revascularization of non-culprit vessels to evaluate its relationship with MACE occurrence in STEMI patients.

Short-term evaluation of patients for a 6-month follow-up limited us with no case of ischemic stroke in this period. Nonetheless, in other studies with a long-term follow-up duration of 12 months in the Australian COPS RCT study and 22 months in COLCOT study, some other predictable outcomes such as stroke, deep vein thrombosis or pulmonary thromboemboly, atrial fibrillation and urgent revascularization were observed and evaluated. So, for future researches, we suggest longer period follow-ups with a larger sample size to reach much more reliable data.

## Conclusion

Finally, we conclude that low-dose prescription of colchicine in ACS patients decreases the rate of major adverse cardiac events in 6 months after a cardiovascular event. Also, the survival rates significantly improved more in colchicine group than in placebo.

## Data Availability

The dataset analyzed during the current study are available from the corresponding author on reasonable request.
